# Assessing the Big Five personality traits using real-life static facial images

**DOI:** 10.1038/s41598-020-65358-6

**Published:** 2020-05-22

**Authors:** Alexander Kachur, Evgeny Osin, Denis Davydov, Konstantin Shutilov, Alexey Novokshonov

**Affiliations:** 1 Artificial Intelligence LLC (AIPictor), BP Mirland, 2-ya Khutorskaya ul. 38Ас15, Moscow, 127287 Russia; 2 National Research University Higher School of Economics, Department of Psychology, International Laboratory of Positive Psychology of Personality and Motivation, Myasnitskaya ul. 20, Moscow, 101000 Russia; 3 Open University for the Humanities and Economics, Department of Psychology and Pedagogy, Nizhegorodskaya ul. 32s4, Moscow, 109029 Russia; 4 BestFitMe Ltd, 67 Grosvenor St, Mayfair, London, W1K 3JN United Kingdom

**Keywords:** Human behaviour, Computer science

## Abstract

There is ample evidence that morphological and social cues in a human face provide signals of human personality and behaviour. Previous studies have discovered associations between the features of artificial composite facial images and attributions of personality traits by human experts. We present new findings demonstrating the statistically significant prediction of a wider set of personality features (all the Big Five personality traits) for both men and women using real-life static facial images. Volunteer participants (N = 12,447) provided their face photographs (31,367 images) and completed a self-report measure of the Big Five traits. We trained a cascade of artificial neural networks (ANNs) on a large labelled dataset to predict self-reported Big Five scores. The highest correlations between observed and predicted personality scores were found for conscientiousness (0.360 for men and 0.335 for women) and the mean effect size was 0.243, exceeding the results obtained in prior studies using ‘selfies’. The findings strongly support the possibility of predicting multidimensional personality profiles from static facial images using ANNs trained on large labelled datasets. Future research could investigate the relative contribution of morphological features of the face and other characteristics of facial images to predicting personality.

## Introduction

A growing number of studies have linked facial images to personality. It has been established that humans are able to perceive certain personality traits from each other’s faces with some degree of accuracy^1–4^. In addition to emotional expressions and other nonverbal behaviours conveying information about one’s psychological processes through the face, research has found that valid inferences about personality characteristics can even be made based on static images of the face with a neutral expression^5–7^. These findings suggest that people may use signals from each other’s faces to adjust the ways they communicate, depending on the emotional reactions and perceived personality of the interlocutor. Such signals must be fairly informative and sufficiently repetitive for recipients to take advantage of the information being conveyed^8^.

Studies focusing on the objective characteristics of human faces have found some associations between facial morphology and personality features. For instance, facial symmetry predicts extraversion^9^. Another widely studied indicator is the facial width to height ratio (fWHR), which has been linked to various traits, such as achievement striving^10^, deception^11^, dominance^12^, aggressiveness^13–16^, and risk-taking^17^. The fWHR can be detected with high reliability irrespective of facial hair. The accuracy of fWHR-based judgements suggests that the human perceptual system may have evolved to be sensitive to static facial features, such as the relative face width^18^.

There are several theoretical reasons to expect associations between facial images and personality. First, genetic background contributes to both face and personality. Genetic correlates of craniofacial characteristics have been discovered both in clinical contexts^19,20^ and in non-clinical populations^21^. In addition to shaping the face, genes also play a role in the development of various personality traits, such as risky behaviour^22–24^, and the contribution of genes to some traits exceeds the contribution of environmental factors^25^. For the Big Five traits, heritability coefficients reflecting the proportion of variance that can be attributed to genetic factors typically lie in the 0.30–0.60 range^26,27^. From an evolutionary perspective, these associations can be expected to have emerged by means of sexual selection. Recent studies have argued that some static facial features, such as the supraorbital region, may have evolved as a means of social communication^28^ and that facial attractiveness signalling valuable personality characteristics is associated with mating success^29^.

Second, there is some evidence showing that pre- and postnatal hormones affect both facial shape and personality. For instance, the face is a visible indicator of the levels of sex hormones, such as testosterone and oestrogen, which affect the formation of skull bones and the fWHR^30–32^. Given that prenatal and postnatal sex hormone levels do influence behaviour, facial features may correlate with hormonally driven personality characteristics, such as aggressiveness^33^, competitiveness, and dominance, at least for men^34,35^. Thus, in addition to genes, the associations of facial features with behavioural tendencies may also be explained by androgens and potentially other hormones affecting both face and behaviour.

Third, the perception of one’s facial features by oneself and by others influences one’s subsequent behaviour and personality^36^. Just as the perceived ‘cleverness’ of an individual may lead to higher educational attainment^37^, prejudice associated with the shape of one’s face may lead to the development of maladaptive personality characteristics (i.e., the ‘Quasimodo complex’^38^). The associations between appearance and personality over the lifespan have been explored in longitudinal observational studies, providing evidence of ‘self-fulfilling prophecy’-type and ‘self-defeating prophecy’-type effects^39^.

Fourth and finally, some personality traits are associated with habitual patterns of emotionally expressive behaviour. Habitual emotional expressions may shape the static features of the face, leading to the formation of wrinkles and/or the development of facial muscles.

Existing studies have revealed the links between objective facial picture cues and general personality traits based on the Five-Factor Model or the Big Five (BF) model of personality^40^. However, a quick glance at the sizes of the effects found in these studies (summarized in Table 1) reveals much controversy. The results appear to be inconsistent across studies and hardly replicable^41^. These inconsistencies may result from the use of small samples of stimulus faces, as well as from the vast differences in methodologies. Stronger effect sizes are typically found in studies using composite facial images derived from groups of individuals with high and low scores on each of the Big Five dimensions^6–8^. Naturally, the task of identifying traits using artificial images comprised of contrasting pairs with all other individual features eliminated or held constant appears to be relatively easy. This is in contrast to realistic situations, where faces of individuals reflect a full range of continuous personality characteristics embedded in a variety of individual facial features.

**Table 1:** Effect sizes found in studies that used integral facial images to predict personality traits.

	*N*_*photo*_	*N*_*rater*_	Effect size, *r*				
			E	A	C	N	O
**Artificial feature manipulation**							
Walker & Vetter, 2016, Study 3	30	160	0.23	0.27	0.12	0.36	0.24
**Composite facial images**							
Little & Perrett, 2007	20	40	0.19	0.06	0.17	0.11	0.10
Penton-Voak *et al*., Study 2	20	42	0.56	0.24	0.63	0.37	0.47
Kramer & Ward, 2010	20	131	0.78	0.37	0.06	0.28	−0.11
**Realistic photographs**							
Penton-Voak *et al*. 2006, Study 1	294	10	0.24	−0.01	0.06	0.17	0.18
Qui *et al*. 2015, Selfies	123	8	0.02	0.06	0.10	0.07	0.21
Borkenau *et al*., 2009	149	24	0.34	0.04	0.16	0.07	0.11
Naumann *et al*., 2009, Standard	123	6	0.39	−0.11	−0.03	0.17	0.17
Naumann *et al*., 2009, Spontaneous	123	6	0.42	0.20	0.12	0.18	0.35
**Machine learning models**							
Hu *et al*., 2017, Male faces	405	n/a	0.16	0.11	0.21	0.15	0.14
Hu *et al*., 2017, Female faces	429	n/a	0.11	0.02	−0.06	−0.06	0.00

Note: N_photo_ – number of stimulus faces, N_rater_ – number of human raters per photograph, E – extraversion, A – agreeableness, C – conscientiousness, N – neuroticism, O – openness. Effect sizes are given as reported by the authors where available or calculated using meta-analytic formulae^26^.

Studies relying on photographic images of individual faces, either artificially manipulated^2,42^ or realistic, tend to yield more modest effects. It appears that studies using realistic photographs made in controlled conditions (neutral expression, looking straight at the camera, consistent posture, lighting, and distance to the camera, no glasses, no jewellery, no make-up, etc.) produce stronger effects than studies using ‘selfies’^25^. Unfortunately, differences in the methodologies make it hard to hypothesize whether the diversity of these findings is explained by variance in image quality, image background, or the prediction models used.

Research into the links between facial picture cues and personality traits faces several challenges. First, the number of specific facial features is very large, and some of them are hard to quantify. Second, the effects of isolated facial features are generally weak and only become statistically noticeable in large samples. Third, the associations between objective facial features and personality traits might be interactive and nonlinear. Finally, studies using real-life photographs confront an additional challenge in that the very characteristics of the images (e.g., the angle of the head, facial expression, makeup, hairstyle, facial hair style, etc.) are based on the subjects’ choices, which are potentially influenced by personality; after all, one of the principal reasons why people make and share their photographs is to signal to others what kind of person they are. The task of isolating the contribution of each variable out of the multitude of these individual variables appears to be hardly feasible. Instead, recent studies in the field have tended to rely on a holistic approach, investigating the subjective perception of personality based on integral facial images.

The holistic approach aims to mimic the mechanisms of human perception of the face and the ways in which people make judgements about each other’s personality. This approach is supported by studies of human face perception, showing that faces are perceived and encoded in a holistic manner by the human brain^43–46^. Put differently, when people identify others, they consider individual facial features (such as a person’s eyes, nose, and mouth) in concert as a single entity rather than as independent pieces of information^47–50^. Similar to facial identification, personality judgements involve the extraction of invariant facial markers associated with relatively stable characteristics of an individual’s behaviour. Existing evidence suggests that various social judgements might be based on a common visual representational system involving the holistic processing of visual information^51,52^. Thus, even though the associations between isolated facial features and personality characteristics sought by ancient physiognomists have emerged to be weak, contradictory or even non-existent, the holistic approach to understanding the face-personality links appears to be more promising.

An additional challenge faced by studies seeking to reveal the face-personality links is constituted by the inconsistency of the evaluations of personality traits by human raters. As a result, a fairly large number of human raters is required to obtain reliable estimates of personality traits for each photograph. In contrast, recent attempts at using machine learning algorithms have suggested that artificial intelligence may outperform individual human raters. For instance, S. Hu and colleagues^40^ used the composite partial least squares component approach to analyse dense 3D facial images obtained in controlled conditions and found significant associations with personality traits (stronger for men than for women).

A similar approach can be implemented using advanced machine learning algorithms, such as artificial neural networks (ANNs), which can extract and process significant features in a holistic manner. The recent applications of ANNs to the analysis of human faces, body postures, and behaviours with the purpose of inferring apparent personality traits^53,54^ indicate that this approach leads to a higher accuracy of prediction compared to individual human raters. The main difficulty of the ANN approach is the need for large labelled training datasets that are difficult to obtain in laboratory settings. However, ANNs do not require high-quality photographs taken in controlled conditions and can potentially be trained using real-life photographs provided that the dataset is large enough. The interpretation of findings in such studies needs to acknowledge that a real-life photograph, especially one chosen by a study participant, can be viewed as a holistic behavioural act, which may potentially contain other cues to the subjects’ personality in addition to static facial features (e.g., lighting, hairstyle, head angle, picture quality, etc.).

The purpose of the current study was to investigate the associations of facial picture cues with self-reported Big Five personality traits by training a cascade of ANNs to predict personality traits from static facial images. The general hypothesis is that a real-life photograph contains cues about personality that can be extracted using machine learning. Due to the vast diversity of findings concerning the prediction accuracy of different traits across previous studies, we did not set a priori hypotheses about differences in prediction accuracy across traits.

## Results

### Prediction accuracy

We used data from the test dataset containing predicted scores for 3,137 images associated with 1,245 individuals. To determine whether the variance in the predicted scores was associated with differences across images or across individuals, we calculated the intraclass correlation coefficients (ICCs) presented in Table 2. The between-individual proportion of variance in the predicted scores ranged from 79 to 88% for different traits, indicating a general consistency of predicted scores for different photographs of the same individual. We derived the individual scores used in all subsequent analyses as the simple averages of the predicted scores for all images provided by each participant.

**Table 2:** BF personality trait prediction accuracy for ANN models.

Trait	Gender	ICC	r	ρ	RMSE	MAE
Agreeableness	Men	0.829 [0.802; 0.852]	0.214 [0.129; 0.295]	0.230	1.253 (1.404)	0.964 (1.100)
	Women	0.811 [0.789; 0.832]	0.238 [0.168; 0.304]	0.254	1.234 (1.378)	0.981 (1.090)
Conscientiousness	Men	0.853 [0.830; 0.873]	0.360 [0.281; 0.433]	0.386	1.130 (1.419)	0.889 (1.130)
	Women	0.821 [0.800; 0.841]	0.335 [0.270;0.398]	0.358	1.152 (1.424)	0.897 (1.140)
Extraversion	Men	0.827 [0.800; 0.851]	0.187 [0.102;0.270]	0.202	1.274 (1.338)	1.019 (1.070)
	Women	0.785 [0.760; 0.809]	0.266 [0.198;0.332]	0.288	1.211 (1.407)	0.945 (1.100)
Neuroticism	Men	0.803 [0.773; 0.830]	0.210 [0.125;0.292]	0.220	1.255 (1.408)	0.995 (1.100)
	Women	0.849 [0.830; 0.866]	0.284 [0.216;0.349]	0.295	1.196 (1.453)	0.951 (1.170)
Openness	Men	0.855 [0.832; 0.875]	0.189 [0.104;0.272]	0.224	1.272 (1.382)	0.986 (1.100)
	Women	0.876 [0.860; 0.890]	0.137 [0.067;0.207]	0.161	1.313 (1.443)	1.036 (1.130)

Note: ICC – intraclass correlation of personality ratings of multiple photographs within individuals; r – Pearson correlation between observed and predicted scores; ρ – correlation estimate corrected for attenuation due to measurement unreliability. 95% confidence intervals are given for ICC and r. The root mean square error (RMSE) and mean average error (MAE) of prediction are calculated based on standardized (z) scores; the numbers in parentheses denote the RMSE and MAE values obtained for a random normal distribution of predicted scores.

The correlation coefficients between the self-report test scores and the scores predicted by the ANN ranged from 0.14 to 0.36. The associations were strongest for conscientiousness and weakest for openness. Extraversion and neuroticism were significantly better predicted for women than for men (based on the z test). We also compared the prediction accuracy within each gender using Steiger’s test for dependent sample correlation coefficients. For men, conscientiousness was predicted more accurately than the other four traits (the differences among the latter were not statistically significant). For women, conscientiousness was predicted more accurately, and openness was predicted less accurately compared to the three other traits.

The mean absolute error (MAE) of prediction ranged between 0.89 and 1.04 standard deviations. We did not find any associations between the number of photographs and prediction error.

### Trait intercorrelations

The structure of the correlations between the scales was generally similar for the observed test scores and the predicted values, but some coefficients differed significantly (based on the z test) (see Table 3). Most notably, predicted openness was more strongly associated with conscientiousness (negatively) and extraversion (positively), whereas its association with agreeableness was negative rather than positive. The associations of predicted agreeableness with conscientiousness and neuroticism were stronger than those between the respective observed scores. In women, predicted neuroticism demonstrated a stronger inverse association with conscientiousness and a stronger positive association with openness. In men, predicted neuroticism was less strongly associated with extraversion than its observed counterpart.

**Table 3:** Trait intercorrelations for observed (first line) and predicted (in parentheses) Big Five scores.

Men	C	E	N	O
A	**0.408** (**0.554**)	0.323 (0.344)	**−0.136** (**−0.333**)	**0.201** (**−0.315**)
C	−	0.383 (0.321)	−0.464 (−0.428)	**−0.123** (**−0.401**)
E		−	**−0.416** **(−0.226)**	**0.253** **(−0.090**)
N			−	0.198 (0.129)
**Women**	**C**	**E**	**N**	**O**
A	**0.403** (0**.592**)	0.318 (0.253)	**−0.294** (**−0.570**)	**0.234** (**−0.239**)
C	−	0.348 (0.348)	**−0.505** (**−0.774**)	**−0.084** (**−0.466**)
E		−	−0.395 (−0.360)	**0.277** (0**.141**)
N			−	**0.149** (0**.511**)

Note: all coefficients are significant at p < 0.05. Coefficients that differ for predicted and observed scores based on the z test are shown in bold. The full matrices are given in Supplementary Tables S1 and S2.

**Figure 1:**
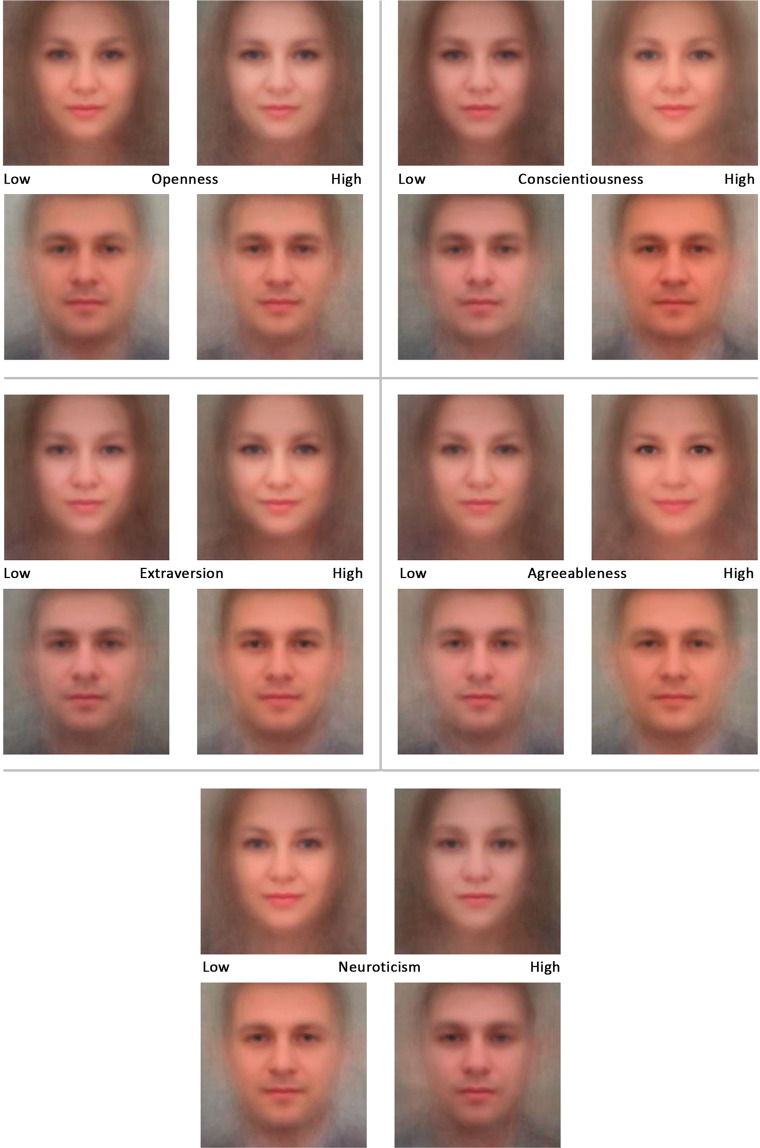
Composite facial images morphed across contrast groups of 100 individuals for each Big Five trait.

To illustrate the findings, we created composite images using Abrosoft FantaMorph 5 by averaging the uploaded images across contrast groups of 100 individuals with the highest and the lowest test scores on each trait. The resulting morphed images in which individual features are eliminated are presented in Fig. 1.

## Discussion

This study presents new evidence confirming that human personality is related to individual facial appearance. We expected that machine learning (in our case, artificial neural networks) could reveal multidimensional personality profiles based on static morphological facial features. We circumvented the reliability limitations of human raters by developing a neural network and training it on a large dataset labelled with self-reported Big Five traits.

We expected that personality traits would be reflected in the whole facial image rather than in its isolated features. Based on this expectation, we developed a novel two-tier machine learning algorithm to encode the invariant facial features as a vector in a 128-dimensional space that was used to predict the BF traits by means of a multilayer perceptron. Although studies using real-life photographs do not require strict experimental conditions, we had to undertake a series of additional organizational and technological steps to ensure consistent facial image characteristics and quality.

Our results demonstrate that real-life photographs taken in uncontrolled conditions can be used to predict personality traits using complex computer vision algorithms. This finding is in contrast to previous studies that mostly relied on high-quality facial images taken in controlled settings. The accuracy of prediction that we obtained exceeds that in the findings of prior studies that used realistic individual photographs taken in uncontrolled conditions (e.g., selfies^55^). The advantage of our methodology is that it is relatively simple (e.g., it does not rely on 3D scanners or 3D facial landmark maps) and can be easily implemented using a desktop computer with a stock graphics accelerator.

In the present study, conscientiousness emerged to be more easily recognizable than the other four traits, which is consistent with some of the existing findings^7,40^. The weaker effects for extraversion and neuroticism found in our sample may be because these traits are associated with positive and negative emotional experiences, whereas we only aimed to use images with neutral or close to neutral emotional expressions. Finally, this appears to be the first study to achieve a significant prediction of openness to experience. Predictions of personality based on female faces appeared to be more reliable than those for male faces in our sample, in contrast to some previous studies^40^.

The BF factors are known to be non-orthogonal, and we paid attention to their intercorrelations in our study^56,57^. Various models have attempted to explain the BF using higher-order dimensions, such as stability and plasticity^58^ or a single general factor of personality (GFP)^59^. We discovered that the intercorrelations of predicted factors tend to be stronger than the intercorrelations of self-report questionnaire scales used to train the model. This finding suggests a potential biological basis of GFP. However, the stronger intercorrelations of the predicted scores can be explained by consistent differences in picture quality (just as the correlations between the self-report scales can be explained by social desirability effects and other varieties of response bias^60^). Clearly, additional research is needed to understand the context of this finding.

We believe that the present study, which did not involve any subjective human raters, constitutes solid evidence that all the Big Five traits are associated with facial cues that can be extracted using machine learning algorithms. However, despite having taken reasonable organizational and technical steps to exclude the potential confounds and focus on static facial features, we are still unable to claim that morphological features of the face explain all the personality-related image variance captured by the ANNs. Rather, we propose to see facial photographs taken by subjects themselves as complex behavioural acts that can be evaluated holistically and that may contain various other subtle personality cues in addition to static facial features.

The correlations reported above with a mean r = 0.243 can be viewed as modest; indeed, facial image-based personality assessment can hardly replace traditional personality measures. However, this effect size indicates that an ANN can make a correct guess about the relative standing of two randomly chosen individuals on a personality dimension in 58% of cases (as opposed to the 50% expected by chance)^61^. The effect sizes we observed are comparable with the meta-analytic estimates of correlations between self-reported and observer ratings of personality traits: the associations range from 0.30 to 0.49 when one’s personality is rated by close relatives or colleagues, but only from −0.01 to 0.29 when rated by strangers^62^. Thus, an artificial neural network relying on static facial images outperforms an average human rater who meets the target in person without any prior acquaintance. Given that partner personality and match between two personalities predict friendship formation^63^, long-term relationship satisfaction^64^, and the outcomes of dyadic interaction in unstructured settings^65^, the aid of artificial intelligence in making partner choices could help individuals to achieve more satisfying interaction outcomes.

There are a vast number of potential applications to be explored. The recognition of personality from real-life photos can be applied in a wide range of scenarios, complementing the traditional approaches to personality assessment in settings where speed is more important than accuracy. Applications may include suggesting best-fitting products or services to customers, proposing to individuals a best match in dyadic interaction settings (such as business negotiations, online teaching, etc.) or personalizing the human-computer interaction. Given that the practical value of any selection method is proportional to the number of decisions made and the size and variability of the pool of potential choices^66^, we believe that the applied potential of this technology can be easily revealed at a large scale, given its speed and low cost. Because the reliability and validity of self-report personality measures is not perfect, prediction could be further improved by supplementing these measures with peer ratings and objective behavioural indicators of personality traits.

The fact that conscientiousness was predicted better than the other traits for both men and women emerges as an interesting finding. From an evolutionary perspective, one would expect the traits most relevant for cooperation (conscientiousness and agreeableness) and social interaction (certain facets of extraversion and neuroticism, such as sociability, dominance, or hostility) to be reflected more readily in the human face. The results are generally in line with this idea, but they need to be replicated and extended by incorporating trait facets in future studies to provide support for this hypothesis.

Finally, although we tried to control the potential sources of confounds and errors by instructing the participants and by screening the photographs (based on angles, facial expressions, makeup, etc.), the present study is not without limitations. First, the real-life photographs we used could still carry a variety of subtle cues, such as makeup, angle, light facial expressions, and information related to all the other choices people make when they take and share their own photographs. These additional cues could say something about their personality, and the effects of all these variables are inseparable from those of static facial features, making it hard to draw any fundamental conclusions from the findings. However, studies using real-life photographs may have higher ecological validity compared to laboratory studies; our results are more likely to generalize to real-life situations where users of various services are asked to share self-pictures of their choice.

Another limitation pertains to a geographically bounded sample of individuals; our participants were mostly Caucasian and represented one cultural and age group (Russian-speaking adults). Future studies could replicate the effects using populations representing a more diverse variety of ethnic, cultural, and age groups. Studies relying on other sources of personality data (e.g., peer ratings or expert ratings), as well as wider sets of personality traits, could complement and extend the present findings.

## Methods

### Sample and procedure

The study was carried out in the Russian language. The participants were anonymous volunteers recruited through social network advertisements. They did not receive any financial remuneration but were provided with a free report on their Big Five personality traits. The data were collected online using a dedicated research website and a mobile application. The participants provided their informed consent, completed the questionnaires, reported their age and gender and were asked to upload their photographs. They were instructed to take or upload several photographs of their face looking directly at the camera with enough lighting, a neutral facial expression and no other people in the picture and without makeup.

Our goal was to obtain an out-of-sample validation dataset of 616 respondents of each gender to achieve 80% power for a minimum effect we considered to be of practical significance (*r* = 0.10 at p < 0.05), requiring a total of 6,160 participants of each gender in the combined dataset comprising the training and validation datasets. However, we aimed to gather more data because we expected that some online respondents might provide low-quality or non-genuine photographs and/or invalid questionnaire responses.

The initial sample included 25,202 participants who completed the questionnaire and uploaded a total of 77,346 photographs. The final combined dataset comprised 12,447 valid questionnaires and 31,367 associated photographs after the data screening procedures (below). The participants ranged in age from 18 to 60 (59.4% women, M = 27.61, SD = 12.73, and 40.6% men, M = 32.60, SD = 11.85). The dataset was split randomly into a training dataset (90%) and a test dataset (10%) used to validate the prediction model. The validation dataset included the responses of 505 men who provided 1224 facial images and 740 women who provided 1913 images. Due to the sexually dimorphic nature of facial features and certain personality traits (particularly extraversion^1,67,68^), all the predictive models were trained and validated separately for male and female faces.

### Ethical approval

The research was carried out in accordance with the Declaration of Helsinki. The study protocol was approved by the Research Ethics Committee of the Open University for the Humanities and Economics. We obtained the participants’ informed consent to use their data and photographs for research purposes and to publish generalized findings. The morphed group average images presented in the paper do not allow the identification of individuals. No information or images that could lead to the identification of study participants have been published.

### Data screening

We excluded incomplete questionnaires (N = 3,035) and used indices of response consistency to screen out random responders^69^. To detect systematic careless responses, we used the modal response category count, maximum longstring (maximum number of identical responses given in sequence by participant), and inter-item standard deviation for each questionnaire. At this stage, we screened out the answers of individuals with zero standard deviations (N = 329) and a maximum longstring above 10 (N = 1,416). To detect random responses, we calculated the following person-fit indices: the person-total response profile correlation, the consistency of response profiles for the first and the second half of the questionnaire, the consistency of response profiles obtained based on equivalent groups of items, the number of polytomous Guttman errors, and the intraclass correlation of item responses within facets.

Next, we conducted a simulation by generating random sets of integers in the 1–5 range based on a normal distribution (µ = 3, σ = 1) and on the uniform distribution and calculating the same person-fit indices. For each distribution, we generated a training dataset and a test dataset, each comprised of 1,000 simulated responses and 1,000 real responses drawn randomly from the sample. Next, we ran a logistic regression model using simulated vs real responses as the outcome variable and chose an optimal cutoff point to minimize the misclassification error (using the R package optcutoff). The sensitivity value was 0.991 for the uniform distribution and 0.960 for the normal distribution, and the specificity values were 0.923 and 0.980, respectively. Finally, we applied the trained model to the full dataset and identified observations predicted as likely to be simulated based on either distribution (N = 1,618). The remaining sample of responses (N = 18,804) was used in the subsequent analyses.

### Big Five measure

We used a modified Russian version of the 5PFQ questionnaire^70^, which is a 75-item measure of the Big Five model, with 15 items per trait grouped into five three-item facets. To confirm the structural validity of the questionnaire, we tested an exploratory structural equation (ESEM) model with target rotation in Mplus 8.2. The items were treated as ordered categorical variables using the WLSMV estimator, and facet variance was modelled by introducing correlated uniqueness values for the items comprising each facet.

The theoretical model showed a good fit to the data (χ^2^ = 147854.68, df = 2335, p < 0.001; CFI = 0.931; RMSEA = 0.040 [90% CI: 0.040, 0.041]; SRMR = 0.024). All the items showed statistically significant loadings on their theoretically expected scales (λ ranged from 0.14 to 0.87, M = 0.51, SD = 0.17), and the absolute cross-loadings were reasonably low (M = 0.11, SD = 0.11). The distributions of the resulting scales were approximately normal (with skewness and kurtosis values within the [−1; 1] range). To assess the reliability of the scales, we calculated two internal consistency indices, namely, robust omega (using the R package coefficientalpha) and algebraic greatest lower bound (GLB) reliability (using the R package psych)^71^ (see Table 4).

**Table 4:** Reliability indices per scale.

	Men		Women	
	Omega	GLB	Omega	GLB
Agreeableness	0.862	0.894	0.878	0.907
Conscientiousness	0.869	0.909	0.876	0.916
Extraversion	0.855	0.899	0.855	0.903
Neuroticism	0.915	0.937	0.924	0.946
Openness	0.714	0.807	0.723	0.812

### Image screening and pre-processing

The images (photographs and video frames) were subjected to a three-step screening procedure aimed at removing fake and low-quality images. First, images with no human faces or with more than one human face were detected by our computer vision (CV) algorithms and automatically removed. Second, celebrity images were identified and removed by means of a dedicated neural network trained on a celebrity photo dataset (CelebFaces Attributes Dataset (CelebA), N > 200,000)^72^ that was additionally enriched with pictures of Russian celebrities. The model showed a 98.4% detection accuracy. Third, we performed a manual moderation of the remaining images to remove images with partially covered faces, those that were evidently photoshopped or any other fake images not detected by CV.

The images retained for subsequent processing were converted to single-channel 8-bit greyscale format using the OpenCV framework (opencv.org). Head position (pitch, yaw, roll) was measured using our own dedicated neural network (multilayer perceptron) trained on a sample of 8 000 images labelled by our team. The mean absolute error achieved on the test sample of 800 images was 2.78° for roll, 1.67° for pitch, and 2.34° for yaw. We used the head position data to retain the images with yaw and roll within the −30° to 30° range and pitch within the −15° to 15° range.

Next, we assessed emotional neutrality using the Microsoft Cognitive Services API on the Azure platform (score range: 0 to 1) and used 0.50 as a threshold criterion to remove emotionally expressive images. Finally, we applied the face and eye detection, alignment, resize, and crop functions available within the Dlib (dlib.net) open-source toolkit to arrive at a set of standardized 224 × 224 pixel images with eye pupils aligned to a standard position with an accuracy of 1 px. Images with low resolution that contained less than 60 pixels between the eyes, were excluded in the process.

The final photoset comprised 41,835 images. After the screened questionnaire responses and images were joined, we obtained a set of 12,447 valid Big Five questionnaires associated with 31,367 validated images (an average of 2.59 images per person for women and 2.42 for men).

### Neural network architecture

**Figure 2:**
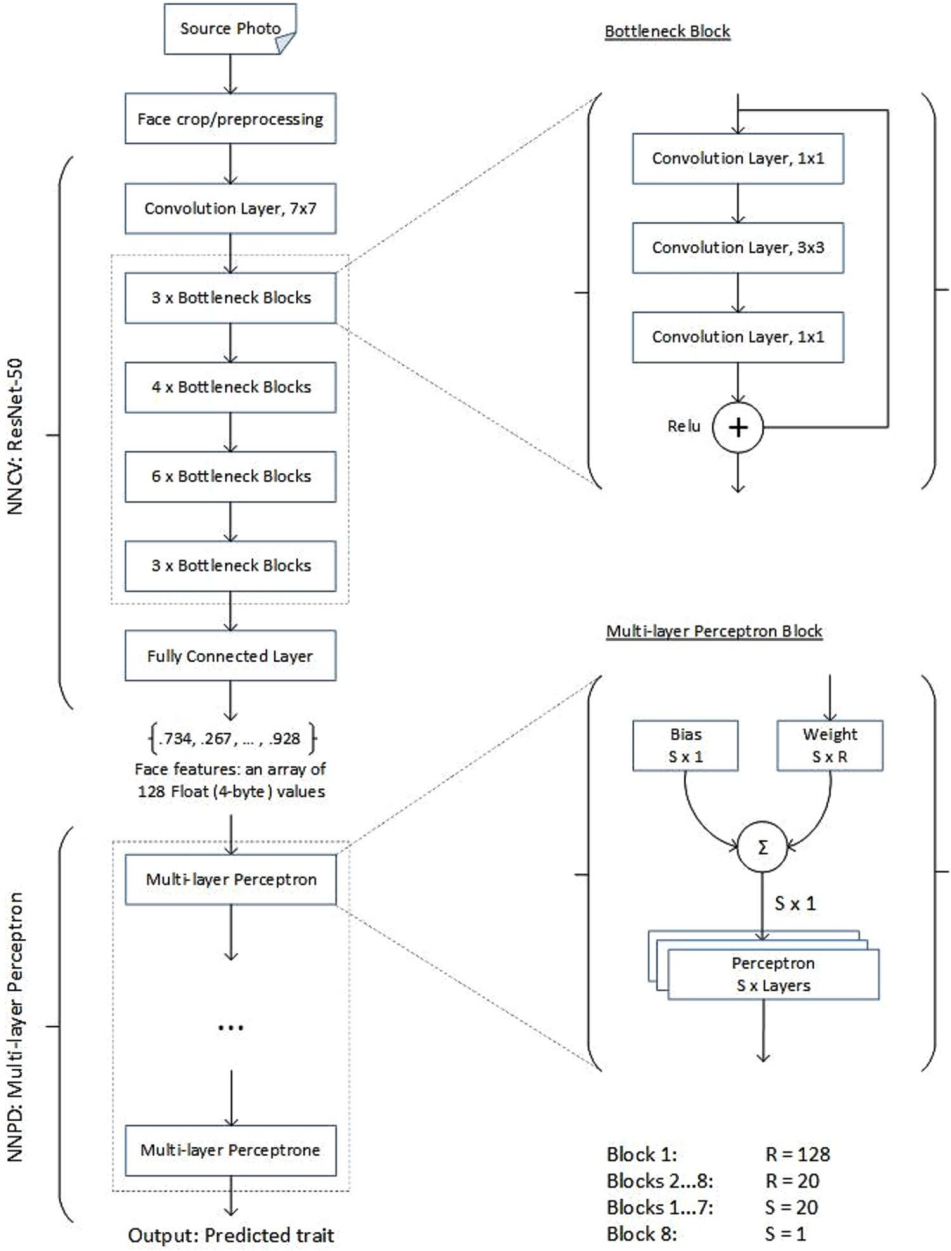
Layer architecture of the computer vision neural network (NNCV) and the personality diagnostics neural network (NNPD).

First, we developed a computer vision neural network (NNCV) aiming to determine the invariant features of static facial images that distinguish one face from another but remain constant across different images of the same person. We aimed to choose a neural network architecture with a good feature space and resource-efficient learning, considering the limited hardware available to our research team. We chose a residual network architecture based on ResNet^73^ (see Fig. 2).

This type of neural network was originally developed for image classification. We dropped the final layer from the original architecture and obtained a NNCV that takes a static monochrome image (224 × 224 pixels in size) and generates a vector of 128 32-bit dimensions describing unique facial features in the source image. As a measure of success, we calculated the Euclidean distance between the vectors generated from different images.

Using Internet search engines, we collected a training dataset of approximately 2 million openly available unlabelled real-life photos taken in uncontrolled conditions stratified by race, age and gender (using search engine queries such as ‘face photo’, ‘face pictures’, etc.). The training was conducted on a server equipped with four NVidia Titan accelerators. The trained neural network was validated on a dataset of 40,000 images belonging to 800 people, which was an out-of-sample part of the original dataset. The Euclidean distance threshold for the vectors belonging to the same person was 0.40 after the training was complete.

Finally, we trained a personality diagnostics neural network (NNPD), which was implemented as a multilayer perceptron (see Fig. 2). For that purpose, we used a training dataset (90% of the final sample) containing the questionnaire scores of 11,202 respondents and a total of 28,230 associated photographs. The NNPD takes the vector of the invariants obtained from NNCV as an input and predicts the Big Five personality traits as the output. The network was trained using the same hardware, and the training process took 9 days. The whole process was performed for male and female faces separately.

## Supplementary information

Supplementary Information.

## Data Availability

The set of photographs is not made available because we did not solicit the consent of the study participants to publish the individual photographs. The test dataset with the observed and predicted Big Five scores is available from the openICPSR repository: 10.3886/E109082V1.
