# Supplementary material for: Assessing the Big Five personality traits using real-life static facial images
Source: Sci Rep. 2020 May 22;10:8487. doi: 10.1038/s41598-020-65358-6 (PMC7244587; doi:10.1038/s41598-020-65358-6)
Supplement: Supplementary file 1 — Supplementary Information. [file 41598_2020_65358_MOESM1_ESM.doc]

**Supplementary Information**

**Assessing the Big Five personality traits using real-life static facial images**

**Alexander Kachur^1^, Evgeny Osin^*2^, Denis Davydov^3^, Konstantin Shutilov^1^, Alexey Novokshonov^1^**

^1^ BestFitMe Ltd, 67 Grosvenor St, Mayfair, London W1K 3JN, United Kingdom

^2^ National Research University Higher School of Economics, Department of Psychology, International Laboratory of Positive Psychology of Personality and Motivation, Moscow, 101000, Russia

^3^ Open University of Humanities and Economics, Department of Psychology and Pedagogy, Moscow, 109029, Russia

* [evgeny.n.osin@gmail.com](mailto:evgeny.n.osin@gmail.com)

**Supplementary Information**

Supplementary Table S1

*Pearson correlations between observed and predicted scores in men (N = 505)*

| Variable | 1 | 2 | 3 | 4 | 5 | 6 | 7 | 8 | 9 |
| --- | --- | --- | --- | --- | --- | --- | --- | --- | --- |
| 1. A predicted |  |  |  |  |  |  |  |  |  |
| 2. C predicted | .55** |  |  |  |  |  |  |  |  |
| 3. E predicted | .34** | .32** |  |  |  |  |  |  |  |
| 4. N predicted | -.33** | -.43** | -.23** |  |  |  |  |  |  |
| 5. O predicted | -.31** | -.40** | -.09* | .13** |  |  |  |  |  |
| 6. A test score | .21** | .15** | .11* | -.08 | .03 |  |  |  |  |
| 7. C test score | .27** | .36** | .16** | -.18** | -.09* | .41** |  |  |  |
| 8. E test score | .10* | .17** | .19** | -.12** | .02 | .32** | .38** |  |  |
| 9. N test score | -.10* | -.22** | -.14** | .21** | .07 | -.14** | -.46** | -.42** |  |
| 10. O test score | -.12** | -.22** | -.14** | .11* | .19** | .20** | -.12** | .25** | .20** |

*Note.* A – agreeableness, C – conscientiousness, E – extraversion, N – neuroticism, O – openness. * indicates *p* < .05. ** indicates *p* < .01.

Supplementary Table S2

*Pearson correlations between observed and predicted scores in women (N = 740)*

| Variable | 1 | 2 | 3 | 4 | 5 | 6 | 7 | 8 | 9 |
| --- | --- | --- | --- | --- | --- | --- | --- | --- | --- |
| 1. A predicted |  |  |  |  |  |  |  |  |  |
| 2. C predicted | .59** |  |  |  |  |  |  |  |  |
| 3. E predicted | .25** | .35** |  |  |  |  |  |  |  |
| 4. N predicted | -.57** | -.77** | -.36** |  |  |  |  |  |  |
| 5. O predicted | -.24** | -.47** | .14** | .51** |  |  |  |  |  |
| 6. A test score | .24** | .20** | .09* | -.20** | -.12** |  |  |  |  |
| 7. C test score | .28** | .34** | .18** | -.28** | -.16** | .40** |  |  |  |
| 8. E test score | .15** | .13** | .27** | -.15** | .06 | .32** | .35** |  |  |
| 9. N test score | -.28** | -.26** | -.21** | .28** | .12** | -.29** | -.50** | -.39** |  |
| 10. O test score | -.07 | -.10** | -.03 | .07* | .14** | .23** | -.08* | .28** | .15** |

*Note.* A – agreeableness, C – conscientiousness, E – extraversion, N – neuroticism, O – openness. * indicates *p* < .05. ** indicates *p* < .01.
